# Significant Turning Point: Common Buzzard (*Buteo buteo*) Exposure to Second-Generation Anticoagulant Rodenticides in the United Kingdom

**DOI:** 10.1021/acs.est.3c09052

**Published:** 2024-03-28

**Authors:** Shinji Ozaki, Paola Movalli, Alessandra Cincinelli, Nikiforos Alygizakis, Alexander Badry, Heather Carter, Jacqueline S. Chaplow, Daniela Claßen, René W. R. J. Dekker, Beverley Dodd, Guy Duke, Jan Koschorreck, M. Glória Pereira, Elaine Potter, Darren Sleep, Jaroslav Slobodnik, Nikolaos S. Thomaidis, Gabriele Treu, Lee Walker

**Affiliations:** † UK Centre for Ecology and Hydrology, Lancaster Environment Centre, Library Avenue, Bailrigg, Lancaster LA1 4AP, United Kingdom; ‡ Naturalis Biodiversity Center, Darwinweg 2, 2333 CR Leiden, Netherlands; § Department of Chemistry “Ugo Schiff”, University of Florence, Via della Lastruccia 3, 50019 Florence, Italy; ∥ Environmental Institute, Okružná 784/42, 97241 Koš, Slovak Republic; ⊥ Department of Chemistry, National and Kapodistrian University of Athens, Panepistimiopolis Zographou, 15771 Athens, Greece; # German Environment Agency (Umweltbundesamt), Wörlitzer Platz 1, 06813 Dessau-Roßlau, Germany; ∇ UK Centre for Ecology and Hydrology, MacLean Bldg, Benson Ln, Crowmarsh Gifford, Wallingford OX10 8BB, United Kingdom

**Keywords:** apex predator, conditional inference trees, effectiveness evaluation, regulatory changes, seasonal fluctuation

## Abstract

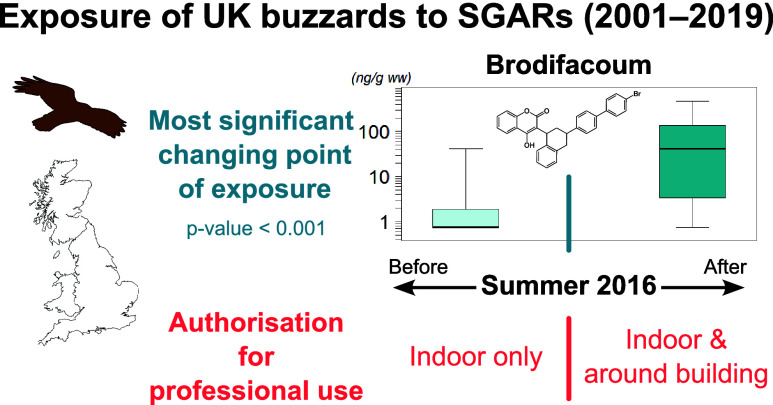

Second-generation anticoagulant rodenticides (SGARs) are widely used to control rodent populations, resulting in the serious secondary exposure of predators to these contaminants. In the United Kingdom (UK), professional use and purchase of SGARs were revised in the 2010s. Certain highly toxic SGARs have been authorized since then to be used outdoors around buildings as resistance-breaking chemicals under risk mitigation procedures. However, it is still uncertain whether and how these regulatory changes have influenced the secondary exposure of birds of prey to SGARs. Based on biomonitoring of the UK Common Buzzard (*Buteo buteo*) collected from 2001 to 2019, we assessed the temporal trend of exposure to SGARs and statistically determined potential turning points. The magnitude of difenacoum decreased over time with a seasonal fluctuation, while the magnitude and prevalence of more toxic brodifacoum, authorized to be used outdoors around buildings after the regulatory changes, increased. The summer of 2016 was statistically identified as a turning point for exposure to brodifacoum and summed SGARs that increased after this point. This time point coincided with the aforementioned regulatory changes. Our findings suggest a possible shift in SGAR use to brodifacoum from difenacoum over the decades, which may pose higher risks of impacts on wildlife.

## Abstract 2

Temporal trend analysis of SGARs in UK buzzards indicated a significant turning point of exposure, the date of which coincided with important regulatory changes for rodenticide use.

## 1 Introduction

Small rodents cause widespread conflict with human interests by transmitting disease and costly damage to crops, food stores, and infrastructure.^1−3^ Anticoagulant rodenticides (ARs) are widely used to control rodent populations to reduce these consequential impacts.^4^ However, the use of ARs has resulted in secondary exposure of various animals, including birds of prey.^5−12^ Exposure of predatory birds to ARs is likely to include feeding on either or both rodenticide “target” and “nontarget” small mammals.^13,14^ Target rodents in the United Kingdom (UK) are typically the brown rat *Rattus norvegicus* and the house mouse *Mus musculus*,^14^ while nontarget rodents are primarily wood mouse *Apodemus sylvaticus* and bank vole *Myodes glareolus*.^15,16^ As rats and mice with resistance to the first-generation AR appeared, more toxic second-generation anticoagulant rodenticides (SGARs) were developed and used,^17,18^ which has resulted in worldwide exposure of wildlife to SGARs and poisoned cases.

Currently, five SGARs are authorized for use in the UK: difenacoum, bromadiolone, brodifacoum, flocoumafen, and difethialone. Among these SGARs, brodifacoum, flocoumafen, and difethialone are more toxic and have longer half-lives in organisms’ tissues than the two others.^10,19^ Only difenacoum and bromadiolone were historically authorized for use “indoor”, “in and around buildings”, and in “open areas”, while the three others were restricted to “indoor” use only in Britain (England, Wales, and Scotland).^17,20,21^ However, the health risk of SGAR-active substances was reviewed by the European Commission (EC) in the second half of the 2000s.^22^ A series of negotiations was then conducted with the UK Competent Authority for biocides, the Health and Safety Executive (HSE), and stakeholder organizations for all professional user groups. Risk mitigation procedures were more precisely defined in the middle of the 2010s with a change in the restrictions on the use of SGARs.^23,24^ Given the development of resistance to bromadiolone and difenacoum within target-rodent populations, the use of products containing brodifacoum, flocoumafen, and difethialone, as chemicals for resistance-breaking and reducing wildlife exposure risk to difenacoum and bromadiolone from rodents with resistance, is now authorized “indoor” and “in and around buildings”, including “sewers” (HSE; https://www.hse.gov.uk/biocides/uk-authorised-biocidal-products.htm; data accessed on 01/01/2024). Meanwhile, the mode, quantity, and frequency of rodenticide use have changed over time. For instance, difenacoum and bromadiolone were widely used, and their quantity increased during the 1990s^25^ and even in the 2000s.^8^ In contrast, the recent trend is to reduce the use of rodenticides.^26^

Given the complex situation with SGAR use, several studies have assessed the temporal trend of exposure of UK wildlife to SGARs (e.g., refs (27−29)), and their results generally show increasing trends in exposure over time. Other studies have demonstrated some significant differences in exposure to SGARs before and after the change in regulation (e.g., refs (30,31)). However, even though exposure of wildlife to SGARs has changed over time, it is still unclear whether and how regulatory changes have influenced the exposure of UK wildlife to SGARs. In the present study, we aimed to determine the exposure of birds of prey to SGARs over time in the UK. We analyze SGARs in livers of the Common Buzzard (*Buteo buteo*) collected in the UK from 2001 to 2019 as a sentinel to assess the general temporal trend of the prevalence and magnitude of each SGAR and to statistically determine potential turning points of exposure during the monitoring period.

## 2 Materials and Methods

### 2.1 Buzzard Sample Collection and Data Preparation

The Common Buzzard (hereafter “buzzard”) is a bird of prey widely distributed across Europe. Inhabiting different habitats, they feed on various prey items, including birds, mammals, reptiles, batrachians, insects, and avian and mammalian carcasses.^32−36^ Their relatively high abundance makes them a favorable raptor for measuring numerous contaminants over large spatial scales.^37^ Buzzards in the UK are nonmigratory and territorial, in contrast to many European populations, and mainly feed on rabbits and small mammals like voles, although their diet is highly dependent on field availability.^38−40^

Seventy-two buzzards found dead or dying in the wild were collected across Britain from 2001 to 2019. Requests for dead bird of prey submissions were made to the public, birdwatchers, rehabilitation centers, and wildlife managers through bird journals, newsletters, and other communications. Carcasses were sent to the Predatory Bird Monitoring Scheme (PBMS) of the UK Centre for Ecology & Hydrology (UKCEH). For each sample, the location and date of sample collection were recorded (for the locations of the samples, see Supporting Information Figure SI1 and Table SI1). All carcasses were subject to a postmortem examination conducted by an experienced wildlife ecologist at UKCEH. After dissection, various tissue samples were stored at −20 °C.

The sex of an individual was determined based on identification of the gonads or bird’s size and plumage. The approximate age was determined from plumage characteristics and assigned following the EURING code.^41^ In the present study, we placed specimens into two age classes: young birds collected in the calendar year of hatching (i.e., juveniles) and older birds (i.e., adults). The sex of two juveniles and the age of one female were unknown, and there was one specimen whose sex and age were unknown (for details, see Table SI2). There was no significant difference in the number of each sex–age category within specimens whose sex and age were identified (*p*-value of the Fisher exact test >0.05). The sample locations were classed into four regions as in the study of Broughton et al.^27^ on SGARs in the Eurasian Sparrowhawk *Accipiter nisus* in the UK: “Scotland”, “northern England” (North West, North East, Yorkshire, & the Humber), “western England & Wales” (West Midlands, South West), and “eastern England” (South West, East of England, London, South East). When liver SGAR residue was detected, birds with hemorrhage in the absence of traumatic injury were considered to be poisoned with SGARs.

### 2.2 SGAR Measurement

0.25 g of each liver was thawed, weighed, dried, and ground with anhydrous sodium sulfate. Each sample was spiked with labeled standards (*d*^5^-bromodialone and *d*^4^-drodifacoum, QMx Laboratories Ltd.). Chloroform/acetone (1:1 v/v) was added to each sample and thoroughly mixed using a vortex. Samples were extracted on a mechanical shaker (Stuart SF1, Bibby Scientific) for 1 h and then centrifuged at 5000 rpm (4696*g* force) for 5 min. The supernatant was transferred to a clean tube. This process was repeated with a clean solvent, but the second time, samples were placed on a mechanical shaker for only 30 min. The combined extract was evaporated to dryness using a parallel evaporator (Büchi Syncore, Switzerland), redissolved in chloroform/acetone (1:1; v/v), and filtered (0.2 mm polytetrafluoroethylene, PTFE, filter). The filtered sample was evaporated to dryness and redissolved in acetone/dichloromethane (1:23; v/v). The sample was refiltered (0.2 mm PTFE filter) and then cleaned using automated size exclusion chromatography (Agilent 1200 HPLC system). The clean extract was evaporated, and the residue was resuspended in chloroform/acetone/acetonitrile (1:1:8; v/v). The extract was further cleaned using solid-phase extraction cartridges (ISOLUTE SI 500 mg, 6 mL). The cartridges were washed with methanol and activated with acetonitrile. The samples were eluted with acetonitrile, and this solvent was then exchanged with mobile phase at the starting composition for the instrument.

Analysis was performed using a “Acquity” UPLC coupled to a triple quadrupole “Xevo TQ-XS” mass spectrometer (Waters Ltd., Wilmslow, UK) interfaced with a “Unispray” source in negative polarity mode and operated with Masslynx software (V.4.2). Analyte separation (1 μL inj. volume) was performed on an Acquity UPLC BEH C18 column (Waters, 1.7 μm particle size, 100 mm × 3 mm I.D.) using a H_2_O/MeOH mobile phase gradient. The analytes were eluted from the column using a program, which mixed different ratios of mobile phase A: 0.77 g/L ammonium acetate in water and mobile phase B: 0.77 g/L ammonium acetate in methanol at a rate of 0.3 mL min^–1^. Gradient elution started from 70% A and 30% B, increased to 65% B in 3 min, and held until 9 min then ramped to 75% B at 12 min and finally to 98% B at 19 min, held for 1.5 min, and then returned to starting conditions.

MS/MS was performed in multiple reaction mode (MRM) using Unispray in negative mode, and characteristic ion fragments were monitored for each compound. Argon was used as the collision gas. Chromatographic peaks were integrated using Masslynx, which was also used to generate linear calibration curves with *R*^2^ > 0.99. The rodenticide standards (Dr Ehrenstorfer, LGC Group, Teddington, UK) were matrix-matched.

The performance of the method was assessed in terms of the limit of detection (LoD), recovery of the internal standards for the analytes, and linearity. Recovery for the total procedure was calculated using the labeled standards. LoD was 1.5 ng/g wet weight (ww) for all compounds except for 3.0 ng/g ww for difethialone. Each liver sample was spiked with deuterated bromadiolone and brodifacoum, and the mean and standard deviation recovery rates for deuterated bromadiolone and brodifacoum were 69.6 ± 8.4 and 71.2 ± 7.0%, respectively.

### 2.3 Data Analysis

#### 2.3.1 Statistical Summary for SGARs

The minimum, median, and maximum liver concentrations were calculated for each of the five active ingredients and the sum of the concentrations of all five SGARs in each specimen (∑SGARs). For each compound, concentrations below the LoD were converted into 0 ng/g ww by assuming that specimens with a concentration below the LoD were not affected by SGARs. The prevalence of each active ingredient and ∑SGARs was estimated by the proportion of specimens, in which the given SGAR was detected.

Co-occurrence of SGARs was assessed by the number of samples containing each combination of these SGARs in the liver of buzzards. We also assessed correlations between the magnitude of bromadiolone, difenacoum, and brodifacoum residues, which are the three SGARs more frequently observed in the liver of predatory birds than flocoumafen and difethialone.^10^ Due to skewed SGAR residue concentration data, we used the nonparametric Spearman’s rank correlation index. The index was calculated for the samples containing these three SGARs and tested for significance.

#### 2.3.2 Temporal Trend of Prevalence and Magnitude of SGARs

The temporal trend of the prevalence and magnitude of SGARs in the liver were separately modeled using logistic and linear regressions, respectively. For both regressions, modeling was carried out for bromadiolone, difenacoum, brodifacoum, and ∑SGARs, and age, sex, region, temporal trend over years, and seasonal fluctuation within years were considered as explanatory variables. The collection date was converted into the midpoint of the month of collection. The month-based time trend was then applied as the temporal trend over years (i.e., the months January to December 2001 were converted into months 1 to 12, and the 12 months in 2002 were converted into months 13–24, etc.). The seasonal fluctuations within years were integrated into models by sine and cosine terms, which explain multiple sine waves,^42,43^ based on the midpoint of 12 months of the collection date, assuming that the phase and amplitude of the seasonal relationship were the same every year. Given the small number of samples compared to the monitoring period, we added only one sine and one cosine term, the combination of which explains one peak and one trough for each year.

For the logistic regression, we used 68 specimens whose sex and age were identified among the 72 buzzards collected (Table SI2). Birds in which the active ingredient residue was detected were given a value of 1, while birds with no detected residue were given a value of 0. This binary response was analyzed with the aforementioned explanatory variables. The significance of the explanatory variables was assessed with the likelihood ratio (LR) test. First, the full models with and without the seasonal trend function (i.e., sine and cosine terms) were compared with the LR test. P-values <0.05 were taken as statistically significant. Then, other explanatory variables were selected by the stepwise selection method^44^ from the full model with or without seasonality, depending on the previous LR test. After checking the assumptions of the selected logistic model, the proportion of the deviance explained by the given model was calculated as a pseudo-*R*^2^ (*R*_D_^2^).^44^

For the magnitude of SGARs, only birds with a detected SGAR residue value were used for the linear regression to avoid confounding the prevalence (i.e., whether they are contaminated or not) and magnitude (i.e., what concentrations). Bromadiolone, difenacoum, brodifacoum, and ∑SGAR residue concentrations were logarithmically transformed, and the same analytical process was applied to select significant explanatory variables using the F-test instead of the LR test. After checking the assumptions of the linear model, the coefficient of determination (*R*^2^) was calculated.

#### 2.3.3 Analysis for Potential Turning Points of Exposure to SGARs

The question about potential turning points for the impact of SGARs on buzzards was assessed by using conditional inference trees (CITs).^45^ The CIT is a recursive binary partitioning analysis for assessing significant univariate splits over all possible splitting variables and all possible splitting points within a variable. The most significant splitting variable or point separating the response values into two groups is selected. This step is recursively performed on the two-split data until no significant difference is observed. Permutation tests were applied for the splitting tests,^46^ and *p*-values were adjusted by the Bonferroni correction.

The CIT was applied to bromadiolone, difenacoum, brodifacoum, and ∑SGARs. We used concentrations of SGARs with 0 values (i.e., values below the LoD) as exposure, which resulted from combination effects of the prevalence and magnitude of SGARs on buzzards. For bromadiolone, difenacoum, and brodifacoum, we used half of the LoD for 0 values. For ∑SGARs, values below the LoD (i.e., 0) were replaced with half of the minimum detected residue value, because no LoD was defined. The month-based across-year trend and age (binary), sex (binary), and collection region (four classes) were applied to the analysis of splitting variables. We also applied this analysis to the number of detected SGARs in one specimen to assess whether and when the number of detected SGARs changed over time.

All statistical analyses were computed using the statistical software R (ver. 4.3.1).^47^ The logistic and linear regression were carried out with the “glm” and “lm” functions in the “stats” package. CIT was carried out with the “ctree” function in the “partykit” package.

## 3 Results

### 3.1 General Summary for SGAR Residues in UK Buzzards

Overall, 62 of the total 72 specimens (86.1%) had detectable residues of one or more SGARs (Table 1). Bromadiolone and difenacoum were detected in 47 (65.3%) and 52 (72.2%) specimens, respectively. Brodifacoum was detected in 30 specimens (41.7%), while flocoumafen and difethialone were detected in two (2.8%) and three specimens (4.2%), respectively. Forty-eight buzzards (66.7%) had more than one detectable residue. The median values of the two dominant SGARs (i.e., bromadiolone and difenacoum) and ∑SGARs were 4.1, 5.7, and 21.8 ng/g ww, respectively, whereas the median values of the others were below the LoD. The maximum ∑SGAR value was 474 ng/g ww. The maximum value of brodifacoum showed a similar value (463.2 ng/g ww), while the maximum value of the others was 100–150 ng/g ww, except for flocoumafen (40 ng/g ww). Only two of the 72 buzzards (2.8%) showed hemorrhage in the absence of traumatic injury and were considered to be poisoned by SGARs. One of them was collected in 2006 with 55.7, 71.4, 4.5, and 131.6 ng/g ww of bromadiolone, difenacoum, brodifacoum, and ∑SGAR residues, respectively. The other was collected in 2018 with 7.3, 86.7, 6.3, and 100.3 ng/g ww of these SGAR residues, respectively.

**Table 1:** Summary for the Magnitude and Prevalence of the Five SGAR-Active Ingredients and Summed SGARs (∑SGARs)^a^

		bromadiolone	difenacoum	brodifacoum	difethialone	flocoumafen	∑SGARs
magnitude (ng/g ww)	minimum	ND	ND	ND	ND	ND	ND
	median	4.1	5.7	ND	ND	ND	21.8
	maximum	104.8	136.7	463.2	146.8	39.9	474.4
prevalence (number of samples)	nondetected	25	20	42	69	70	10
	detected	47	52	30	3	2	62
	% of detected	65.3%	72.2%	41.7%	4.2%	2.8%	86.1%

a The minimum, median, and maximum values, as well as the number of samples with detected and nondetected SGAR, are represented. Nondetected residue values are represented by “ND”.

**Figure 1:**
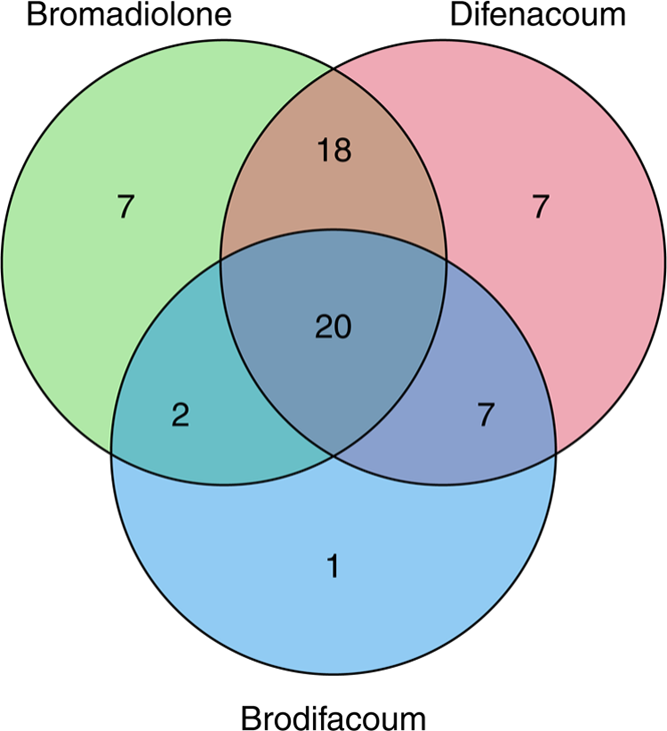
Venn diagram for co-occurrence of bromadiolone, difenacoum, and brodifacoum in the liver of buzzards (*n* = 62) collected across the UK from 2001 to 2019.

Among the 62 specimens having more than one detectable SGAR, 20 had detectable bromadiolone, difenacoum, and brodifacoum in their livers (Figure 1). Seven other specimens had detectable bromadiolone residues, and seven others had detectable difenacoum residues. Brodifacoum residues were detected in 30 specimens, and 29 of them were coexposed to either or both bromadiolone and difenacoum. Flocoumafen was detected in two specimens among the 20 with the three SGARs discussed above. Difethialone was detected in two specimens with the three SGARs and in one specimen with brodifacoum. The minimum, median, and maximum numbers of detected SGARs in one specimen were zero, two, and four, respectively.

Correlations among the magnitudes of bromadiolone, difenacoum, and brodifacoum were assessed in the 20 specimens, in which all three SGARs were detected, and no significant correlation was observed. Spearman correlation index was 0.27 between bromadiolone and difenacoum, 0.16 between difenacoum and brodifacoum, and −0.01 between bromadiolone and brodifacoum (for a visual representation, see Figure SI2).

### 3.2 Temporal Trends of the Prevalence and Magnitude of SGAR Residues

**Figure 2:**
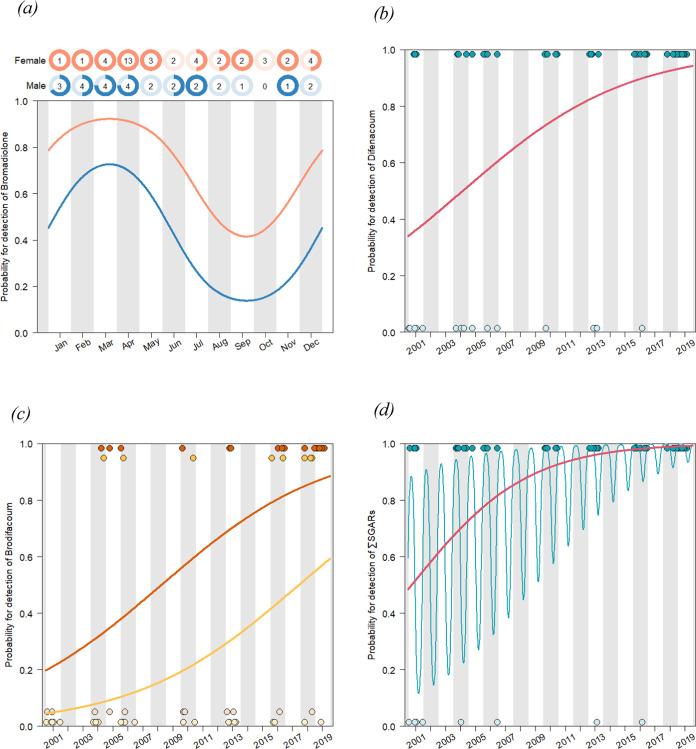
Prevalence of bromadiolone (a), difenacoum (b), brodifacoum (c), and ∑SGARs (d) in the liver of 68 UK buzzards collected from 2001 to 2019 in relation to their collection date. The continuous lines represent prevalence modeled with the logistic regression, and each point represents the collection date of buzzards with SGAR detected (located at 1) and nondetected (located at 0). For bromadiolone, the proportion of buzzards with detected SGAR residues is represented by the pie chart with the number of collected samples. Females and males are distinguished by red and blue colors, respectively. For brodifacoum, adults and juveniles (<1 year) are distinguished by brown and yellow colors, respectively. For ∑SGARs, the blue line represents the modeled prevalence with seasonal and across-year trends, and the red bold line represents only the across-year trend by fixing prevalence in June for each year.

Prevalence of bromadiolone showed a significant within-year seasonal fluctuation (*p*-value of LR test between the model and the same model without seasonal fluctuation = 0.003) and was significantly higher in female buzzards than in males (*p*-value = 0.01; *R*_D_^2^ = 0.181) (Figure 2a). Higher prevalence was observed from late winter to early spring (predicted probability for the detection of bromadiolone in females: 0.92; males: 0.73), and the prevalence trough was in autumn (females: 0.42; males: 0.14). However, there was no significant across-year temporal trend for bromadiolone. In contrast, the prevalence of difenacoum significantly increased over time from 0.34 in January 2001 to 0.94 in December 2019 (*p*-value <0.001; *R*_D_^2^ = 0.184) (Figure 2b). Prevalence of brodifacoum also significantly increased over time (*p*-value <0.001) and was significantly higher in adults than in juveniles (*p*-value = 0.005; *R*_D_^2^ = 0.294) from 0.20 and 0.04 in January 2001 to 0.88 and 0.59 in December 2019, respectively (Figure 2c). Prevalence of ∑SGARs showed both seasonal and across-year temporal trends (*p*-value = 0.01 and <0.001, respectively; *R*_D_^2^ = 0.352) (Figure 2d). The seasonal prevalence peak of ∑SGARs was in late winter–early spring, and its trough was in early autumn (for a visual representation of the seasonal trend, see Figure SI3). Prevalence also increased over years from 0.51 in June 2001 to 0.99 in June 2019.

**Figure 3:**
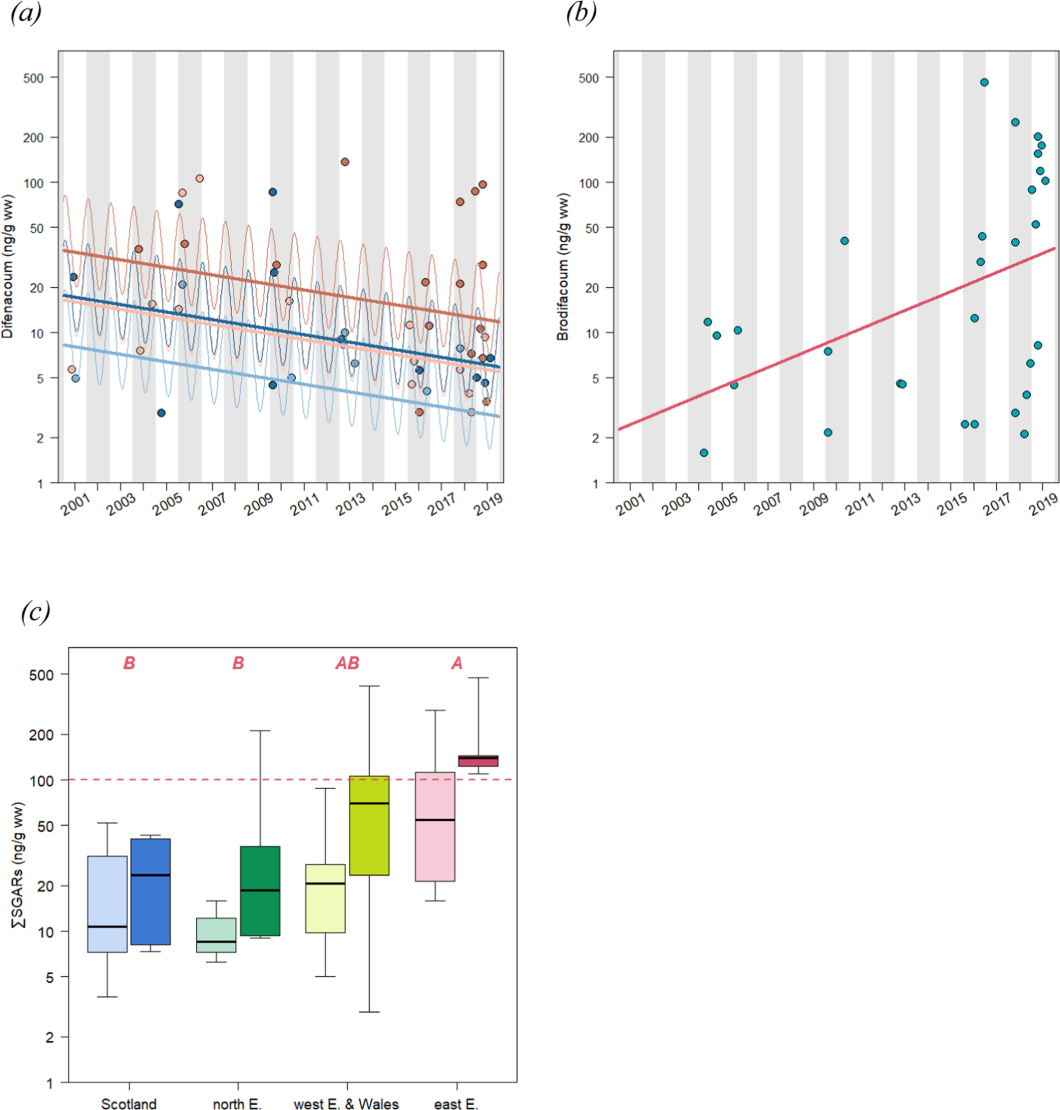
Concentrations of difenacoum (*n* = 49; a), brodifacoum (*n* = 30; b), and ∑SGARs residues (*n* = 59; c) in the liver of UK buzzards collected from 2001 to 2019. For difenacoum and brodifaoum, each point represents a concentration of the given SGAR in an individual in relation to their collection date, and the continuous lines represent modeled values with the linear model. For difenacoum, the thin lines represent modeled values with seasonal and across-year trends, and the bold lines represent only the across-year trend by fixing concentrations in June for each year. Females and males are distinguished by red and blue colors, while adults and juveniles (<1 year) are distinguished by their dark and clear colors, respectively. For ∑SGARs, concentrations are represented by area, and significant differences are represented by different letters. Adults (right side of each area) and juveniles (left side) are distinguished by dark and clear colors, respectively.

Concentrations of bromadiolone showed no significant seasonal or across-year temporal trends (for a visual representation, see Figure SI4a). They did not significantly differ between sexes or age classes, neither. Difenacoum residues decreased over time (*p*-value = 0.02; *R*^2^ = 0.375) (Figure 3a) and were higher in the winter and lower in the late summer (*p*-value = 0.01; for the seasonal trend within a year, see Figure SI4b). Moreover, difenacoum residues were significantly higher in females than males (*p*-value = 0.02) and higher in adults than in juveniles (*p*-value = 0.008). In contrast, brodifacoum residues showed only an increasing trend over time (*p*-value = 0.01; *R*^2^ = 0.196) (Figure 3b). Summed SGARs showed no significant seasonal or across-year temporal trend (for a visual representation of ∑SGARs over years, see Figure SI4c) but significantly differed between the four areas (*p*-value <0.001) and between adults and juveniles (*p*-value <0.001; *R*^2^ = 0.358) (Figure 3c). Summed SGARs were significantly higher in adults (geometric mean of ∑SGARs: 47.7 ng/g ww) than in juveniles (22.5 ng/g ww). Summed SGARs were also significantly higher in East England (geometric mean: 77.6 ng/g ww) than in Scotland (15.6 ng/g of ww) and Central England (16.4 ng/g ww) from the Tukey HDR test. Buzzards from Wales and West England showed intermediate values (35.1 ng/g ww), which were not significantly different from the others.

### 3.3 Potential Turning Points of Exposure to SGARs over Time

**Figure 4:**
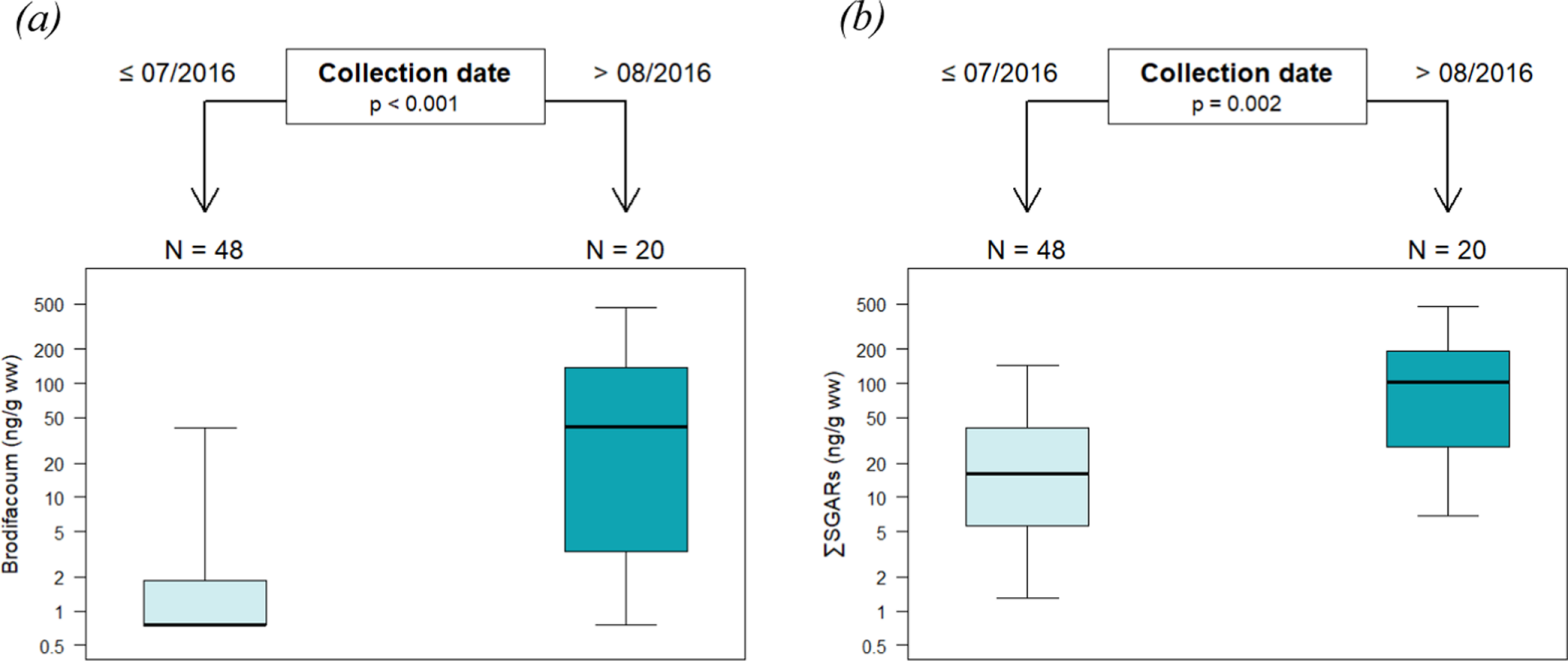
Conditional inference trees representing significant factors influencing exposure of 68 UK buzzards from 2001 to 2019 to brodifacoum (a) and ∑SGARs (b). The upper part of graphics represents significant factors among sex, age, and collection area and date and their conditions distinguishing exposure with *p*-values after the Bonferroni correction. The lower part represents the number of samples and their concentrations of the given SGAR residues in each category.

Among the factors tested by the CIT, the month-based temporal trend significantly distinguished exposure to both brodifacoum and ∑SGARs into two groups (Figure 4). Exposure to brodifacoum and ∑SGARs most significantly differed between after August 2016 (*n* = 20) and before (*n* = 48) (*p*-value after Bonferroni correction <0.001 and = 0.002, respectively). The median values for brodifacoum until July and after August 2016 were 0 and 41.7 ng/g ww, while the median values for ∑SGARs were 16.0 and 100.3 ng/g ww, respectively. The proportion of the specimen number of the two groups significantly differed from 1:1 (chi-squared = 11.5, df = 1, *p*-value <0.001). The number of detected SGARs in one specimen most significantly differed between after September 2013 (*n* = 26; median = 3) and before (*n* = 42; median = 2) (*p*-value <0.001). However, the number of two groups is not significantly different from 1:1 (chi-squared = 3.76, df = 1, *p*-value = 0.052).

## 4 Discussion

### 4.1 Exposure of UK Buzzards to SGARs

Difenacoum and bromadiolone, and to a lesser extent, brodifacoum, were the three dominant SGARs observed in our buzzards. These three active ingredients have also been observed in other UK wild animals, such as sparrowhawk,^27^ barn owls *Tyto alba*,^30^ polecats *Mustela putorius*,^29^ and foxes *Vulpes vulpes*.^48^ The quantity and frequency of use of each SGAR in the UK may reflect these results. For example, approximately 85 tonnes of rodenticidal products were used on Scottish grassland and fodder farms in 2021, and products containing bromadiolone, difenacoum, and brodifacoum accounted for 61, 33, and 4% of this weight, respectively.^26^ Although no data is available for the recent SGAR use in the other countries of Britain, we suspect that the brodifacoum use would also be lower than the two others like Scotland. However, despite its lower usage, brodifacoum showed the highest maximum residue concentration of the five SGARs, probably due to its long half-lives in the rodent’s liver and high accumulation capacity. For example, laboratory mice showed a longer half-life of brodifacoum in the liver (more than 300 days) than bromadiolone (30 days) or difenacoum (60 days).^10^ Moreover, brodifacoum and difenacoum exhibit greater potential for bioaccumulation with high log octanol/water partition coefficients than bromadiolone.^18^ The results of co-occurrence and correlation between SGARs also confirm the long half-life of brodifacoum in livers of rodents or, possibly, birds of prey. From the nonsignificant results of our correlation test, it is assumed that the three SGARs would not be from the same prey. However, brodifacoum was consistently observed with other SGARs, contrary to bromadiolone or difenacoum. Each UK rodenticide product contains one SGAR, except for some products containing both difenacoum and bromadiolone (HSE; https://www.hse.gov.uk/biocides/uk-authorised-biocidal-products.htm; data accessed on 01/01/2024). It is therefore assumed that brodifacoum remains for a long time in the tissue of rodents and predators and is accumulated in predators throughout their lives.

Prevalence of SGAR residues in buzzards in this study (86.1% for ∑SGARs) was higher than recent UK common kestrels *Falco tinnunculus* (67%)^28^ and comparable to barn owls (78–94% annually)^30^ and red kites *Milvus milvus* (82–100% annually) in Britain.^31,49^ In contrast, the magnitude of SGARs in buzzards was lower than in barn owls and red kites: about a quarter of barn owls and the majority of red kites had more than 100 ng/g ww of ∑SGAR in the liver. The proportion of poisoning cases in our buzzards (2.8% of the samples) was between those for barn owls (with 0% of poisoning cases in most years)^30^ and red kites (5–32%).^51^ Liver concentrations associated with rodenticide poisoning vary greatly between species and individuals within species^5,11,50^ due to inter- and intraspecific variations in several physiological mechanisms, such as hepatic vitamin K epoxide reductase activity, metabolisms, anticoagulant binding capacity, and/or blood clotting.^51^ To study the relationship between exposure and effects on free-living animals, it is also necessary to consider other factors, such as different toxicity of various contaminants, their interactions, and sampling biases of the study (e.g., ref (52)). Estimating the impacts of SGARs on the health and population dynamics of wild raptors requires further in-depth studies.

### 4.2 Seasonal and Across-Year Temporal Trend of SGARs and Turning Points

Our study observed seasonal fluctuations in the prevalence of bromadiolone, difenacoum, and ∑SGARs in buzzards. Seasonal variation of SGAR exposure has also been reported in several studies. For example, British polecats collected in the 1990s showed a higher prevalence of ∑SGARs during the first half of the year (January–June) than the second half,^53,54^ and the authors assumed that feeding on rats in winter might result in such a seasonal pattern. Although not significantly different, English red kites from 1989 to 2007 showed a peak prevalence of SGARs in April and May.^55^ Other studies discussed the relationship between the seasonal variations in exposure risk of predator animals to ARs and in their diet.^56,57^ The influence of the seasonal diet change on exposure to SGARs is unclear for our samples and may be a challenge for further studies. However, a recent survey showed that more than 60% of annual SGAR use in Scottish farms was focused on autumn and winter.^26^ Although a recent trend is unclear, ARs were mostly used in winter and spring away from buildings in British game estates in the 1990s.^58^ Increased use of SGARs from autumn to spring could explain our findings of seasonal variation of SGARs in UK buzzards.

Meanwhile, the prevalence of brodifacoum increased over time but did not vary seasonally, probably due to its long half-life in the body of prey, which might obscure seasonal fluctuations. A more recent study on UK polecats from 2013 to 2016 showed no significant seasonal variation in ∑SGARs,^29^ and the authors argued that the risk of recent exposure to SGARs did not vary seasonally compared to that in the 1990s. To deal with our small number of samples over the 19-year monitoring period, we integrated within-year fluctuations into our models by assuming that cycles of the prevalence and magnitude of SGARs would be similar every year. Consequently, the possible variability of seasonal fluctuation over the monitoring period cannot be assessed with our limited sample size. However, given the increasing trend of the prevalence of brodifacoum in UK buzzards, we suspect that seasonal variation in the prevalence of ∑SGARs might differ over the years; seasonal variation might be observed in early years of monitoring but has obscured recent years due to the prevalence of brodifacoum.

Like prevalence, the magnitude of brodifacoum also increased over years, contrary to the decreasing trend of the magnitude of difenacoum. As the magnitude of ∑SGARs did not significantly change over time, our results indicate a recent increase in the relative contribution of brodifacoum to ∑SGAR residues in buzzards. Such a contrast in the trends among SGARs was also observed in recent UK barn owls^30^ and red kites.^49^ A possible shift in the usage practices of products with different active ingredients was suggested in these studies. The mass of bromadiolone and difenacoum used in Scottish farms declined by 32 and 41% between 2017 and 2021, respectively, whereas application of products containing brodifacoum remained at a similar level to 2013,^26^ then increased by 16% from 2017 to 2021. The reasons for these changes were unclear in that survey, but if similar changes in use extended to the other parts of Britain, this might, at least in part, explain the changes in the residue magnitude of each ingredient observed in buzzards.

Such an increase in the use of brodifacoum may result in an increased exposure risk in wildlife. Our analysis indicates that exposure to brodifacoum and ∑SGARs was most significantly distinguished between before and after August 2016. This time period in 2016 does not represent the middle point of our monitoring period nor, given the results of our chi-squared test, the middle point of the data, which means that ∑SGARs suddenly increased after this period. Therefore, the summer of 2016 can be considered as a significant “turning point” of exposure to brodifacoum and ∑SGARs. Historically, the regulatory framework concerning SGAR-containing products gradually changed in the 2010s with the introduction of a stewardship scheme designed to promote best practices in professional use.^24^ For example, the deadline for ceasing the use of AR products with prestewardship labels for professional outdoor use was set on the first of June 2016,^23^ the date of which coincides with this turning point indicated by our analysis. Meanwhile, the number of detected SGARs per specimen increased over the years. However, given the nonsignificant results of the chi-squared test, a time point indicated by CIT may be the middle point of its constant increasing trend. From these results, we suspect that wildlife was contaminated by brodifacoum before the regulatory changes despite its indoor-only use restrictions for almost 30 years.^17^ However, the magnitude of exposure to this active ingredient significantly increased after the regulatory change was implemented.

In contrast, no significant time point was identified for bromadiolone and difenacoum. The decreasing trend of the magnitude of difenacoum may be compensated for by the increasing trend of its prevalence. These results suggest the possibility of various exposure sources with a low quantity of difenacoum. It is now recognized that AR contamination is widely spread in various wild animals, such as small passerines or invertebrates, that are potentially exposed to ARs by ingesting baits, rodent carcasses, feces and/or soil-bound residues.^59−61^ In the UK, the insectivorous small mammal European hedgehog *Erinaceus europaeus* collected during 2004–2006^62^ and the bird-eating raptor sparrowhawk during 1995–2015^27^ showed a high prevalence of SGARs, particularly difenacoum (47.5 and 72.2%, respectively). Given the generalist diet of buzzards,^38,40^ such various foods might be potentially additional sources of exposure.

An increase in brodifacoum in raptors was also observed in the Canary Islands (Spain), despite the reclassification of anticoagulant rodenticides applied from the first of March 2018 (Commission Regulation (EU) 2016/1179), restricting the accessibility of rodenticide baits with >30 ppm of anticoagulant for amateur use.^63^ Similarly, the measures on the use of SGARs only in bait box since 2013 did not reduce exposure of raptors to SGARs in France.^64^ In the United States, although the accessibility of SGARs to nonprofessional applicators is not allowed since the middle of the 2010s, the prevalence of brodifacoum in red-tailed hawks remained almost 100%, and the others increased.^65^ In contrast, an increase in brodifacoum and a decrease in bromadiolone in terrestrial raptors were reported in Western Canada after the regulation measures restricting outdoor use of brodifacoum.^11^ In the last case, only bromadiolone was permitted for outdoor use by licensed operators, which might lead a switch in sales of bromadiolone and brodifacoum.^11^ These outcomes and ours illustrate that a change in places (indoor/outdoor) for professional use or following changes in quantity of use may significantly influence the exposure of raptors to SGARs. However, the efficacy of measures may also differ within SGARs, especially between historically widely used SGARs (bromadiolone and difenacoum) and the others.

### 4.3 Environmental and Biological Factors Influencing SGARs in UK Buzzards

Exposure to bromadiolone and difenacoum was higher in female than male buzzards, compared to many other studies. For example, no difference between sexes was reported in various raptor species in France,^64^ California condor *Gymnogyps californianus* in California,^66^ polecats in the UK,^29^ passerine birds in Germany,^61^ and kestrels in the UK^28^ and Spain.^67^ In other studies, males showed significantly higher ∑SGARs than females, such as sparrowhawks in the UK^27^ and barn owls in Canada.^68^ The prevalence of bromadiolone in the common weasel *Mustela nivalis* from southern Europe was higher in males than females.^69^ These authors mentioned differences in diet and home range between sexes as well as the transfer of contaminants to bird eggs as possible reasons for higher exposure patterns in males than females. Although female buzzards are slightly larger than males, to our knowledge, no clear difference in the diet or home range has been reported between the sexes.^38−40,70^ Nonetheless, one possible reason might be the feeding ecology of buzzards during the incubation period. Females carry out most of the incubation, which is in spring in the UK, while males hunt prey, eat its head, and provide the remaining body to the nest.^39,40^ Given that more than half of SGARs are accumulated and remain in the liver of intoxicated rodents,^71^ ingesting different body parts of prey might result in different exposure levels between the two sexes during the incubation and chick-rearing. However, eating the head is usually observed for big prey like rabbits,^38^ and it is uncertain whether such behavior occurs also for small mammal prey. Moreover, female red kites and kestrels also spend most of their incubation time, and males of these species also provide food to the nest,^39,72,73^ but it remains a question whether different body parts of prey are preferentially shared between sexes. On the other hand, laboratory male white leghorn chickens (*Gallus gallus*) demonstrated higher metabolic ability for warfarin, one of the first-generation anticoagulant rodenticides, than females.^74^ Although there are no data on the difference between sexes in the metabolic ability of wild raptors for SGARs, a similar trend in pharmacokinetics may be expected.

Age was also an important factor for the prevalence or magnitude of SGARs in buzzards, and adults showed a higher prevalence or magnitude than juveniles. These results concur with some other studies (e.g., refs (28,29)). In our results, age influenced difenacoum and brodifacoum concentrations, both of which have a higher potential for bioaccumulation than bromadiolone.^18^ Given the difference in the quantity and frequency of SGAR used by humans and their historical context (e.g., refs (24,26,58)), it is reasonable that animals have accumulated difenacoum and met more opportunities to be exposed to brodifacoum with increasing age.

The magnitude of ∑SGAR in buzzards was significantly higher in eastern England than in northern England or Scotland. Broughton et al.^27^ also demonstrated high SGAR concentrations in sparrowhawks from eastern England, where urbanization and intensive agriculture coverage was higher than in the other parts. Moreover, Roos et al.^28^ observed a positive relationship between the prevalence of SGARs in UK kestrels and the percentage of arable cereals, confirming high SGAR usage in arable farms. The proportion of rats in the diet of barn owls increased with the degree of urbanization,^75^ and the percentage of urban area was a good indicator for the prevalence of AR residue in foxes.^76^ Another possible reason for the high magnitude in eastern England may be rodent resistance to SGARs. Rodents with resistance have spread widely since the 1950s and now cover most of the southern part of England.^17,27,77^ These rodents might accumulate higher SGAR concentrations in their body and, consequently, increase high exposure risks of their predators. Although the region did not statistically explain the prevalence or magnitude of the other SGARs, their patterns might differ among regions. However, our limited data could not allow an assessment of in-depth variations in exposure. Further studies are needed to elucidate entangled interdependent relationships between SGAR residues in raptors, their ecology and physiology, and the spatial distribution and SGAR residues in prey, including target rodents resistant to SGARs.

In conclusion, bromadiolone and difenacoum were predominant SGARs in UK buzzards in the last two decades. However, both the prevalence and magnitude of brodifacoum, a more toxic SGAR than bromadiolone and difenacoum, increased over the years. The level of exposure to brodifacoum particularly increased after the regulatory changes in 2016. Despite the implementation of the stewardship scheme and its promotion of best practice and application of SGARs among professional users (e.g., CRRU^78^), increasing or stable use of brodifacoum might limit the intended reduction in SGAR exposure risk to wildlife, or even increase this risk, because of its longer half-life within the body of prey and potentially higher toxicity than bromadiolone and difenacoum. However, exposure patterns also depend on factors other than SGAR uses by humans, such as the ecology and diet of predators and rodents. Further studies on the difference in exposure to SGARs between species and the spatial distribution of rodents, particularly rodents resistant to SGARs, are expected to clarify the time trend of exposure of wildlife in general.

## Supporting Information Available

Locations of sample collection (Figure SI1); pairwise plot for the correlations between bromadiolone, difenacoum, and brodifacoum residue concentrations (Figure SI2); graphical representation of the seasonal fluctuations within a year of the prevalence of ∑SGARs in the liver of UK buzzards (Figure SI3); graphical representation of the magnitude of bromadiolone and ∑SGAR residues in the liver of UK buzzards over years, as well as the magnitude of difenacoum residues in the liver of UK within a year (Figure SI4); details of the number of samples by year and by sex, age class, or area (Table SI1); and summary for the number of buzzards used in this study (Table SI2) (PDF)

## Abbreviations

**AR**: anticoagulant rodenticide
**LoD**: limit of detection
**SGAR**: second-generation anticoagulant rodenticide
**UK**: United Kingdom
