# Supplementary material for: Significant Turning Point: Common Buzzard (Buteo buteo) Exposure to Second-Generation Anticoagulant Rodenticides in the United Kingdom
Source: Environ Sci Technol. 2024 Mar 28;58(14):6093–104. doi: 10.1021/acs.est.3c09052 (PMC11008253; doi:10.1021/acs.est.3c09052)
Supplement: Supplementary file 1 — es3c09052_si_001.pdf [file es3c09052_si_001.pdf]

Supporting Information for:

Significant Turning Point: Common Buzzard (*Buteo buteo*) Exposure to Second-Generation Anticoagulant Rodenticides in the United Kingdom

Shinji Ozaki*[1], Paola Movalli[2], Alessandra Cincinelli[3], Nikiforos Alygizakis[4,5], Alexander Badry[6], Heather Carter[1], Jacqueline S. Chaplow[1], Daniela Claßen[6], René W. R. J. Dekker[2], Beverley Dodd[1], Guy Duke[7], Jan Koschorreck[6], M. Glória Pereira[1], Elaine Potter[1], Darren Sleep[1], Jaroslav Slobodnik[4], Nikolaos S. Thomaidis[5], Gabriele Treu[6], and Lee Walker[1]

1 UK Centre for Ecology and Hydrology, Lancaster Environment Centre, Library Avenue, Bailrigg, Lancaster, LA1 4AP, United Kingdom
2 Naturalis Biodiversity Center, Darwinweg 2, 2333 CR Leiden, Netherlands
3 Department of Chemistry "Ugo Schiff", University of Florence, Via della Lastruccia 3, 50019 Florence, Italy
4 Environmental Institute, Okružná 784/42, 97241 Koš, Slovak Republic
5 Department of Chemistry, National and Kapodistrian University of Athens, Panepistimiopolis Zographou, 15771 Athens, Greece
6 German Environment Agency (Umweltbundesamt), Wörlitzer Platz 1, 06813 Dessau-Roßlau, Germany
7 UK Centre for Ecology and Hydrology, MacLean Bldg, Benson Ln, Crowmarsh Gifford, Wallingford, OX10 8BB, United Kingdom

* Corresponding author: Shinji Ozaki
E-mail address: ShiOza@ceh.ac.uk

Table of Contents:

**Supporting Information Figure SI1**	Page S1
**Supporting Information Figure SI2**	Page S2
**Supporting Information Figure SI3**	Page S3
**Supporting Information Figure SI4**	Page S4
**Supporting Information Table SI1**	Page S5
**Supporting Information Table SI2**	Page S6

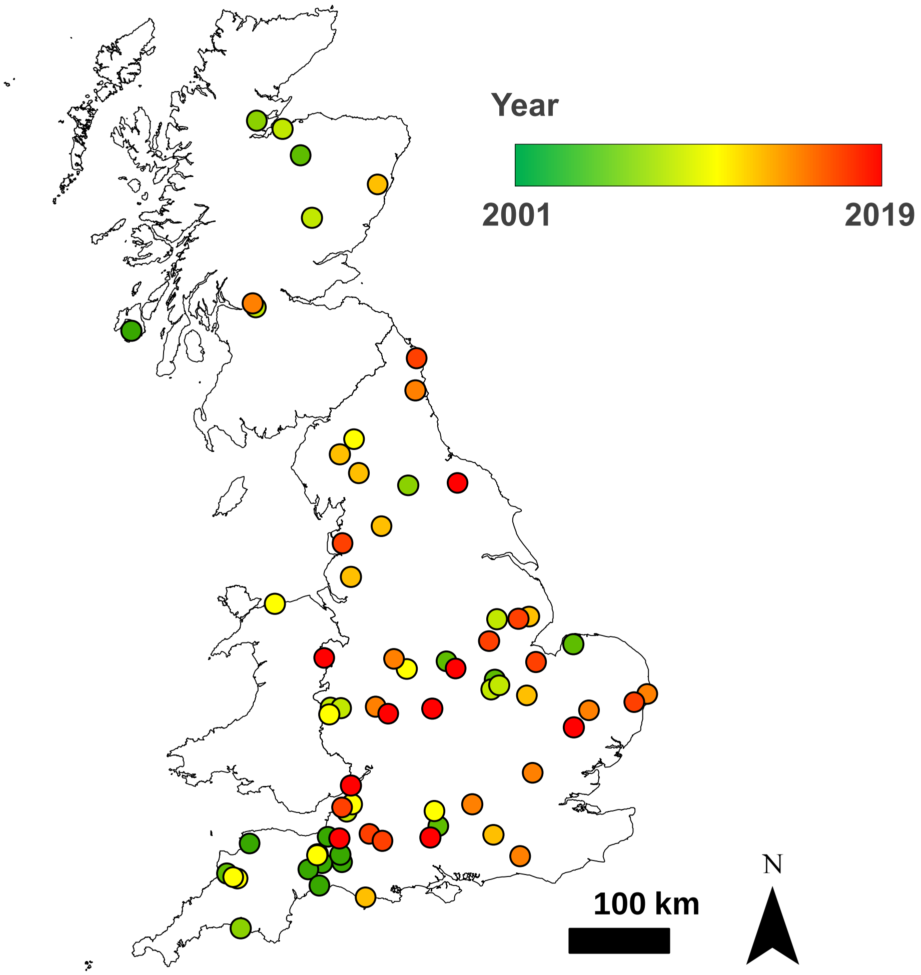

Supporting Information Figure SI1. *Locations of the 72 samples used in the study.*

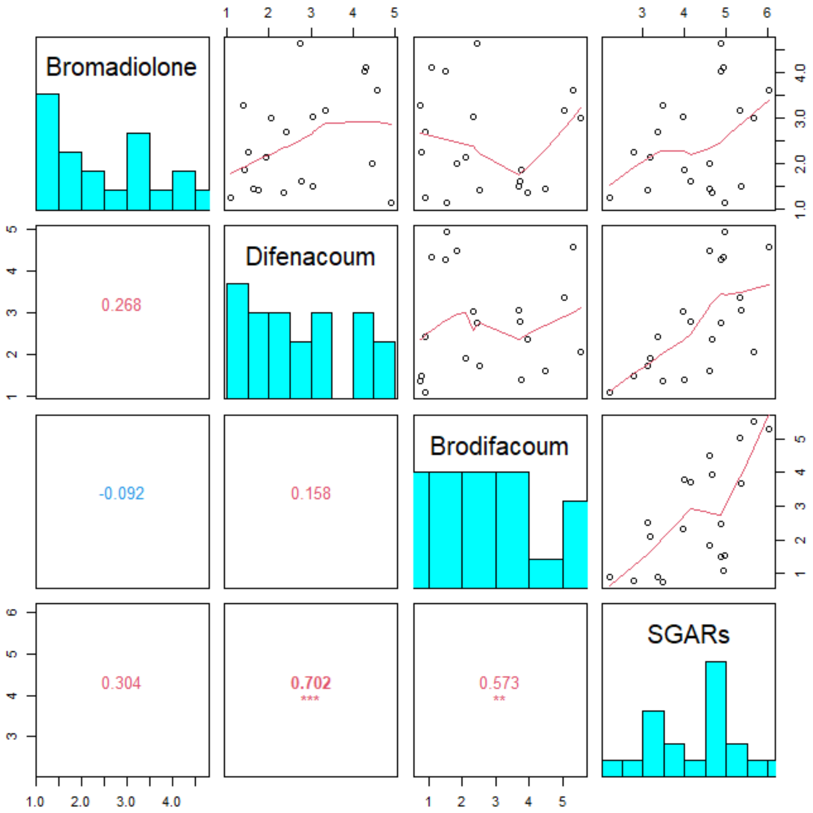

Supporting Information Figure SI2. *Pairwise plot for the Spearman's rank correlation index and graphical representation for correlations between bromadiolone, difenacoum, and brodifacoum residues in the liver of buzzards.*

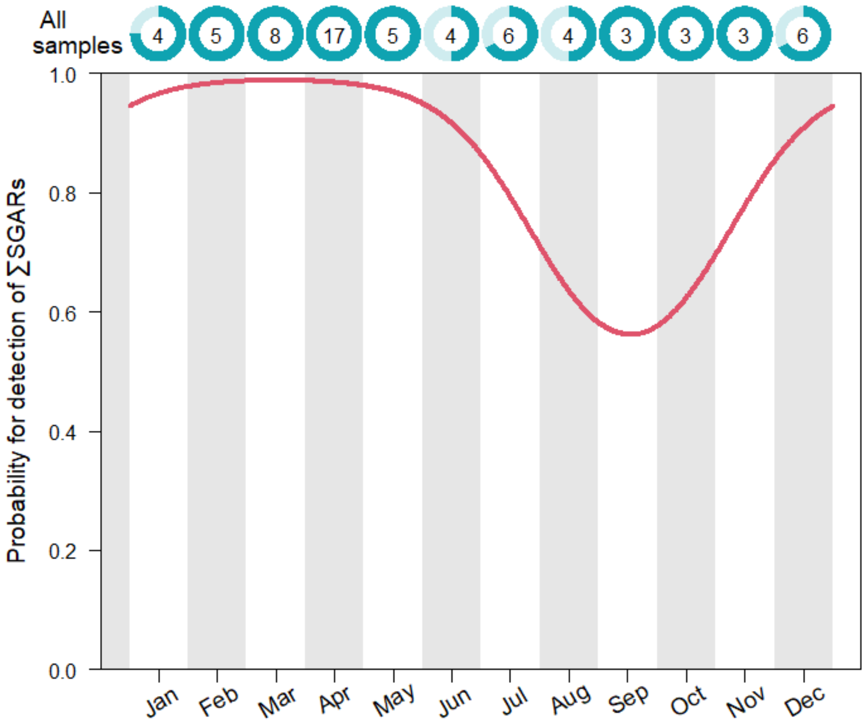

Supporting Information Figure SI3. *Prevalence of ΣSGARs in the liver of 68 UK buzzards collected from 2001 to 2019 in relation to their collection month. The continuous lines represent prevalence modeled with the logistic regression for the middle year of the monitoring period (2011). The proportion of buzzards with detected SGAR residues are represented by the pie chart with the number of collected sample.*

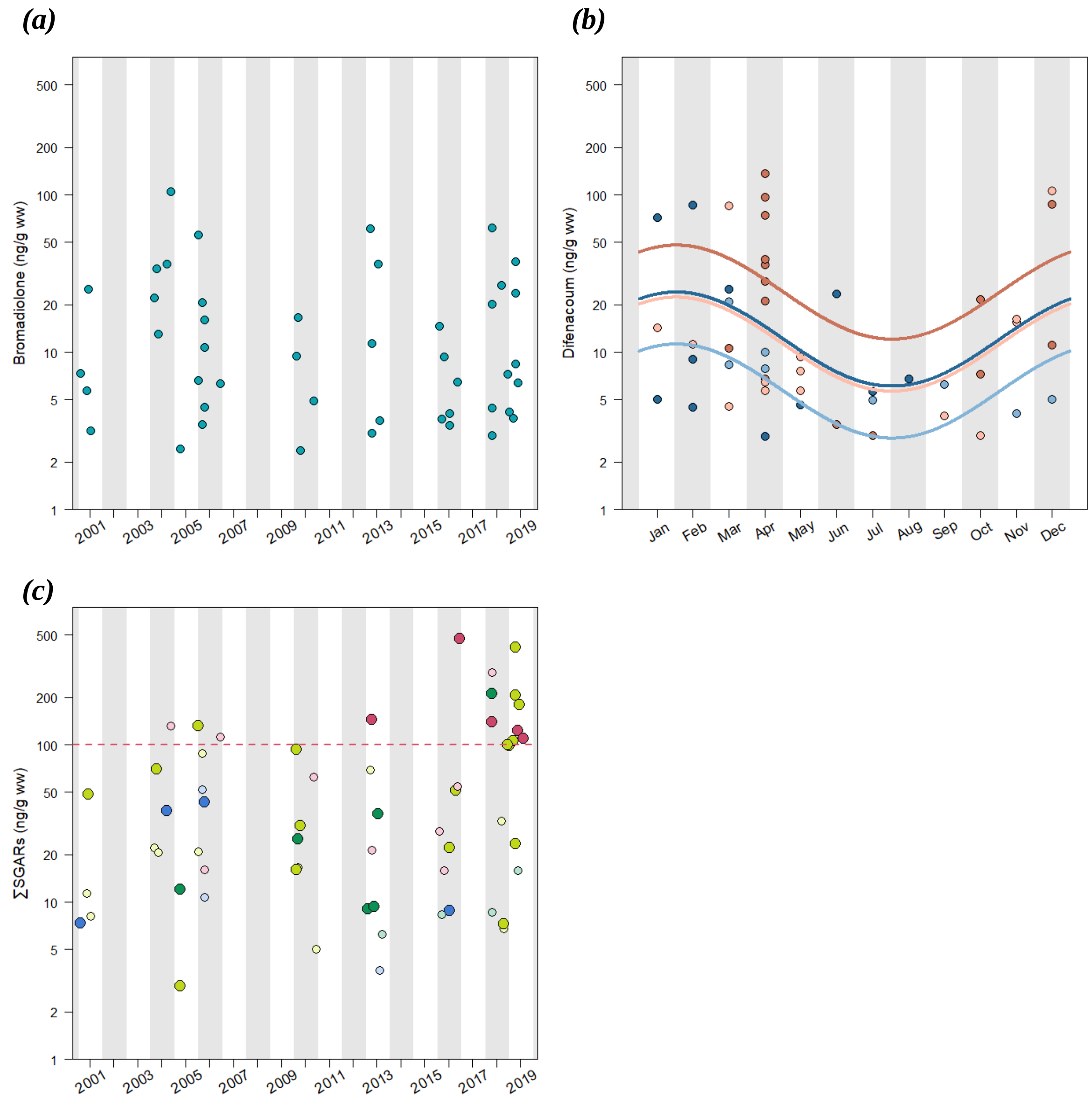

Supporting Information Figure SI4. *Concentrations of bromadiolone (n = 45; a), difenacoum (n = 49; b), and ΣSGARs residues (n = 59; c) in the liver of UK buzzards collected from 2001 to 2019. Each point represents a concentration of the given SGAR in an individual in relation to their collection date (a, c) or collection month (b). For difenacoum, the continuous lines represent modeled values with the linear model for the middle year of the monitoring period (2011). Females and males are distinguished by red and blue colors, while adults and juveniles (<1 year) are distinguished by their dark and clear colors, respectively. For ΣSGARs, four regions are represented by different colors (red: eastern England; yellow: western England; green: northern England; blue: Scotland). Adults and juveniles (<1 year) are distinguished by the size of points (big point: adult; small point: juvenile). The red dashed line represents the concentration of 100 ng/g ww.*

Supporting Information Table S1. *Details of the number of samples by year and by sex, age class, or area. (N. Eng: northern England; W. Eng.: western England and Wales; E. Eng.: eastern England)*

**Year**		**2001**	**2002**	**2003**	**2004**	**2005**	**2006**	**2007**	**2008**	**2009**	**2010**
**Sample number**		**9**	**0**	**0**	**6**	**3**	**9**	**0**	**0**	**0**	**9**
**Sex**	*Female*	*4*			*5*	*0*	*6*				*3*
	Male	*5*			*1*	*2*	*3*				*4*
	Unknown	*0*			*0*	*1*	*0*				*2*
**Age class**	*Adult*	*2*			*2*	*2*	*2*				*4*
	Juvenile	*7*			*4*	*1*	*7*				*4*
	Unknown	*0*			*0*	*0*	*0*				*1*
**Area**	*Scotland*	*1*			*1*	*1*	*3*				*0*
	N. Eng.	*0*			*0*	*1*	*0*				*1*
	W. Eng.	*8*			*3*	*1*	*3*				*7*
	E. Eng.	*0*			*2*	*0*	*3*				*1*

**Year**		**2011**	**2012**	**2013**	**2014**	**2015**	**2016**	**2017**	**2018**	**2019**
**Sample number**		**0**	**0**	**9**	**0**	**0**	**9**	**0**	**9**	**9**
**Sex**	*Female*			*4*			*6*		*8*	*6*
	Male			*5*			*3*		*1*	*3*
	Unknown			*0*			*0*		*0*	*0*
**Age class**	*Adult*			*4*			*5*		*4*	*8*
	Juvenile			*5*			*4*		*4*	*1*
	Unknown			*0*			*0*		*1*	*0*
**Area**	*Scotland*			*1*			*1*		*0*	*0*
	N. Eng.			*4*			*1*		*2*	*1*
	W. Eng.			*1*			*2*		*4*	*6*
	E. Eng.			*3*			*5*		*3*	*2*

Supporting Information Table SI2. *Summary for the number of buzzards used in this study.*

	**Adult**	**Juvenile**	**Unknown**	**Total**
**Female**	18	23	1	42
**Male**	15	12	0	27
**Unknown**	0	2	1	3
**Total**	33	37	2	**72**
